# The Effect of Cold Plasma Pretreatment on Water-Suspended Herbs Measured in the Content of Bioactive Compounds, Antioxidant Activity, Volatile Compounds and Microbial Count of Final Extracts

**DOI:** 10.3390/antiox10111740

**Published:** 2021-10-30

**Authors:** Ewelina Pogorzelska-Nowicka, Monika Maria Hanula, Marta Brodowska-Trębacz, Elżbieta Górska-Horczyczak, Urszula Jankiewicz, Tomasz Mazur, Monika Marcinkowska-Lesiak, Andrzej Półtorak, Agnieszka Wierzbicka

**Affiliations:** 1 Department of Technique and Food Product Development, Institute of Human Nutrition Sciences, Warsaw University of Life Sciences-SGGW, Nowoursynowska 159 c Street, 02-776 Warsaw, Poland; monika_hanula@sggw.edu.pl (M.M.H.); marta_brodowska@sggw.edu.pl (M.B.-T.); elzbieta_gorska_horczyczak@sggw.edu.pl (E.G.-H.); monika_marcinkowska_lesiak@sggw.edu.pl (M.M.-L.); andrzej_poltorak@sggw.edu.pl (A.P.); agnieszka_wierzbicka@sggw.edu.pl (A.W.); 2 Department of Biochemistry and Microbiology, Institute of Biology, Warsaw University of Life Sciences—SGGW, 159 Street, 02-776 Warsaw, Poland; urszula_jankiewicz@sggw.edu.pl; 3 Academic Centre for Materials and Nanotechnology, AGH University of Science and Technology, 30 Mickiewicza Av., 30-059 Krakow, Poland; tmazur@agh.edu.pl

**Keywords:** herbs, cold plasma, green extraction, antioxidants, volatile compounds

## Abstract

Cold plasma is a new technology of promising potential to use as a part of technological extraction lines constructed to implement green chemistry solutions or simply to reduce resources in solvent-based extraction lines. The present study was undertaken to verify the effect of nitrogen cold plasma pre-treatment conducted for 8 min (20 kHz) on the content of antioxidants, antioxidant activity, the profile of volatile compounds, microbial count, pH and color measured in herb extracts (12 herbs: *Echinacea purpurea; Salvia officinalis; Urtica dioica; Polygonum aviculare; Vaccinium myrtillus; Taraxacum officinale; Hypericum perforatum; Achillea millefolium; Sanguisorba officinalis; Leonurus cardiaca; Ballota nigra; Andrographis paniculata*) obtained with its usage. The surface morphology of extracted herbs was examined as well. Herbs used for extraction were ground and suspended in water before cold plasma treatment, which is a novel approach not studied before. Most plasma-treated extracts were characterized by a higher content of polyphenols (11 out of 12). Content of flavonoids and anthocyanins increased in four extracts and in the case of anthocyanins was significantly higher in comparison to control (up to 77%). The antioxidant activity measured at least by one method (ABTS, DPPH, FRAP) was also higher in nine plasma-treated solutions. Moreover, plasma decreased total aerobic bacteria, affected the color and increased pH of the extracts. The surface structure of the plant material after the extraction process was significantly damaged, which probably led to a higher extraction yield of bioactive compounds and in consequence to the higher antioxidant activity of extracts obtained with the cold plasma treatment.

## 1. Introduction

Herbs are widely used in medicine and the food industry, mainly because they contain various bioactive phytochemicals, such as polyphenols, phenolic acids, flavonoids, saponins and curcuminoids [1], which have numerous health beneficial properties. For instance, polyphenols are used to prevent the occurrence of heart diseases, cancers, diabetes, metabolic syndrome and other diseases [2]. Thus, herbs were used for centuries not only as food seasonings but also as a potent remedy for human diseases. The World Health Organization reported that approximately 80% of the world’s human population uses herbs for health maintenance. Moreover, thanks to antioxidant properties they also exhibit high potential as a food preservative. For that purpose, they are added into food either in a native form (fresh or dried and ground) or more frequently as extracts.

Extraction techniques of plant materials used nowadays are still based on the solvent’s usage, which has an environmental impact [3]. Moreover, traditional extraction requires high temperatures and a long time to complete the process. In turn, high temperatures may worsen the qualitative and quantitative characteristics of the obtained extracts. Therefore, proposing a green extraction method of plant materials that will reduce energy consumption, time of the process, solvent usage and post-treatment wastewater has drawn lately considerable scientific attention [3,4].

Cold plasma (CP) is a novel non-thermal technology with a promising application in many industries. Plasma is the fourth state of matter existing in the universe, created by the ionization of gas with the usage of a large amount of energy. The generation of plasma leads to the formation of active chemical species (free radicals, electrons, ultraviolet photons, positive and negative ions, non-excited and excited atoms and molecules). Active species are responsible for the great germicidal properties of plasma treatments.

Reactive compounds created by plasma, oxidate microbial cell membranes while UV radiation degrades DNA, which in turn leads to the inactivation of microorganisms. There are many studies documenting that cold plasma treatment effectively decreases the growth of microorganisms in food [5,6]. In turn, herbs are very often microbiologically contaminated, as most of them are imported from countries of rather poor hygienic in production environments [7]. Previous studies have confirmed that herbs and spices are characterized by high bacterial counts [8]. Thus, it is of utmost importance to use a non-thermal extraction method that would decontaminate the obtained extracts. 

However, it is of great importance to choose the right source of gas for plasma generation. It has been proved that usage of nitrogen as a plasma source led to increasing ruptures of green tea leaves surfaces, which helped to extract more phenolics [9]. Moreover, nitrogen plasma generates very little amount of active oxygen species, protecting in this way bioactive compounds from oxidation in comparison to other gases.

Using plasma as the first step of extraction seems to be a promising alternative for the increased quality of obtained extracts while being cost-effective at the same time. It does not require buying a whole new system of extraction but supports the existing technological lines.

To the best of the authors’ knowledge, available data on the plasma treatment concerns only dry spices or fresh herbs. There is no data on the effect of cold plasma on dry herbs. Moreover, there was found no study on the effect of suspension of spices, herbs, or any dry matter in water before plasma treatment on the antioxidant activity of obtained extracts. It is of key importance taking into consideration research findings that report that plasma acts differently in aqueous solutions than in dry matter [10]. In a water environment plasma is more effective. Thus, the aim of the presented study will be to assess the impact of cold plasma treatment on the structure of herbs as well as on the microbial count, antioxidant activity and volatile compounds profile of twelve different herb extracts obtained suspending herbs in water and further treated with cold plasma and extracted in a water bath.

## 2. Materials and Methods

### 2.1. Extraction Process

The experiment was conducted with the usage of 12 different dry herbs: purple coneflower (*Echinacea purpurea),* Sage (*Salvia officinalis*), Stinging nettle (*Urtica dioica*), Knotweed (*Polygonum aviculare*), Bilberry (*Vaccinium myrtillus*), Dendolin (*Taraxacum officinale*), St. John’s wort (*Hypericum perforatum*), Yarrow (*Achillea millefolium*), Great Burnet (*Sanguisorba officinalis*), Motherwort (*Leonurus cardiaca*), Black Horehound (*Ballota nigra*), Andrographis (*Andrographis paniculata*), which were ground before the extraction process. Herbs were bought from five companies: Dary Natury s.c. (*Salvia officinalis*, *Urtica dioica*, *Polygonum aviculare*, *Taraxacum officinale*, *Hypericum perforatum*, *Achillea millefolium*, *Sanguisorba officinalis*, *Leonurus cardiaca*), NANGA Przemysław Figura s.c. (*Ballota nigra*), Podlaski Ogórd Ziołowy s.c. (*Echinacea purpurea*), Healthylife s.c. (*Andrographis paniculata*) and FLOS s.c. (*Vaccinium myrtillus*). The material was divided into two different groups: the first one was suspended in water using 250 mL glass beakers (1 g in 50 mL), stirred and extracted at 70 °C for 10 min., the second one was suspended in water, stirred and subjected to cold plasma treatment before further extraction in a hot water bath (at 70 °C for 10 min). Extraction in the water bath was performed on samples suspended in water (both for treated and non-treated groups) no additional solvents were used. The surface area of extracts exposed to cold plasma action was 4418 cm^2^. Equal plasma exposure of the entire liquid was ensured using a magnetic stirrer. Plasma activated extracts were prepared using a nonthermal atmospheric pressure plasma jet system (Diener electronic GmbH & Co. KG, Ebhausen, Germany), which consisted of a 20 kHz plasma generator, plasma jet, gas and power conductor. The plasma was generated using an AC power source (220 V) and compressed nitrogen (N2) as the feed gas (flow rate =1 L/min.). Each solution was placed 15 cm below the plasma nozzle and activated by nitrogen plasma for 8 min. Obtained extracts were centrifuged for 5 min at 9327× *g*, filtered through Whatman paper, individually packed for each method and kept at −60 °C until analysis. 

### 2.2. pH Determination

The pH of the extracts was measured using a Five EasyTM pH-meter (MATTLER TOLEDO, Greifensee, Switzerland), previously calibrated in 4.01 and 7.00 buffers at ambient temperature. A glass electrode was placed directly into the extracted sample. For each sample, three measurements were made.

### 2.3. Color Analysis 

The color of the extract was measured in CIE L*a*b* system using Konica Minolta chromameter (CR400. Konica Minolta Inc., Tokyo, Japan), calibrated on a white standard plate. The measurement area was illuminated by a D65 light source. Twelve measurements were taken for each extract.

### 2.4. Total Phenolic Content Analysis

Total phenolic compounds (TPC) content in herb extracts was examined according to the method presented by Singleton and Rossi [11]. Briefly, Folin-Ciocalteu’s reagent, Na_2_CO_3_ and water were mixed with the extract and shaken thoroughly. Then, the mixture was kept in a dark place for 30 min. After this time, the absorbance was measured at a wavelength of 750 nm using a UV-VIS spectrophotometer (UV-1800, Shimadzu Corp., Tokyo, Japan). TPC was expressed as mg of gallic acid equivalent per gram of dry weight.

### 2.5. Total Anthocyanin Analysis

The total anthocyanin content (TA) was performed according to the pH differential method described by Belwal et al. [12]. In brief, an aliquot of herb extract (100 µL) was mixed with potassium chloride (1.5 mL, 0.025 M, pH 1.0), while another was mixed with sodium acetate (1.5 mL, 0.4 M, pH 4.5). The absorbance was measured for both solutions at two wavelengths 520 nm and 700 nm using a UV–VIS spectrophotometer (UV-1800, Shimadzu Corp., 115 VAC, Tokyo, Japan). TA content was expressed as mg of cyanidin 3-glucoside equivalent per gram of dry weight.

### 2.6. Total Flavonoid Analysis

Total flavonoids (TF) content was quantified using the aluminum chloride colorimetric method described by Chang et al., [13]. In brief, herb extract (0.5 mL) was mixed with 0.5 mL of 10% aluminum trichloride (*m*/*v*), 0.1 mL of potassium acetate (1 M) mixture, 1.5 mL of water and then incubated for 30 min at room temperature. After that, the absorbance was checked at 415 nm against the blank using a UV–VIS spectrophotometer (UV-1800, Shimadzu Corp., 115 VAC, Tokyo, Japan). The results were expressed as mg of quercetin equivalent per gram of dry weight (mg QE/g dm). 

### 2.7. DPPH Analysis

The analysis of the free radical-scavenging effect of the antioxidants present in the herb extracts on 2,2-diphenyl-1-picrylhydrazyl (DPPH) radical was determined according to the procedure described by Shimada et al., [14]. In accordance with the methodology, DPPH solution was mixed with herb extracts, shaken and incubated in the dark place for 30 min at room temperature. After that time, the absorbance of the mixture was measured at a wavelength of 520 nm using a UV-VIS spectrophotometer (UV-1800, Shimadzu Corp., 115 VAC, Tokyo, Japan). Results were expressed as mg of ascorbic acid equivalent per gram of dry weight.

### 2.8. FRAP Analysis

FRAP analysis was performed according to the method described by Belwal et al. [12]. FRAP solution was prepared by mixing 2,4,6-Tri(2-pyridyl)-s-triazine (10 mM in 40 mM HCl), ferric chloride (20 mM) and sodium acetate buffer (300 mM, pH 3.6) in a ratio 1:1:10. Then, herb extracts were mixed with FRAP solution and incubated for 15 min in the dark place. After this time, the absorbance of the solutions was measured at 593 nm wavelength using a UV-VIS spectrophotometer (UV-1800, Shimadzu Corp., 115 VAC, Tokyo, Japan). Results were expressed as mg of ascorbic acid equivalent per gram of dry weight.

### 2.9. ABTS Radical Cation Scavenging Activity

The 2,2-anizobis-3-ethylbenzthiazoline-6-sulphonic acid (ABTS) was analyzed according to Belwal’s [12] method. The ABTS mixture (2.45 mM potassium persulfate with 7 mM ABTS salt) was combined with ethanol until an absorbance of 0.7 (at 734 nm). Then, herb extracts (0.1 mL) were added to ABTS solution (2.9 mL), mixed and incubated for 30 min in the dark place. After that, the absorbance was measured at 734 nm wavelength using a UV–VIS spectrophotometer (UV-1800, Shimadzu Corp., 115 VAC, Tokyo, Japan). Obtained results were expressed as mg of ascorbic acid equivalent per gram of dry weight. 

### 2.10. Electronic Nose Analysis

The volatile compounds profiles of herb extracts were analyzed using Heracles II Electronic Nose (Alpha M.O.S., Toulouse, France), equipped with two columns of different polarity (MXT-5 nonpolar and MXT-1701 polar). Main volatile compounds in the samples were detected based on Kovats retention indexes. Analysis parameters were set as follows: injector at 200 °C, oven temperature program 60 °C for 2 s, a 3 °C s^−1^ ramp to 270 °C, isotherm for 30 s at 270 °C, and flame ionization detectors (FID) at 270 °C. Herb extracts (1 mL) were put into 20 mL glass vials and locked with Teflon-silicon rubber caps. Each vial was incubated at 45 °C for 10 min (under an agitation of 8.33 Hz). After that time, the autosampler injected 3500 µL of gas from the samples (headspace) to the columns (with a rate of 125 mL s^−1^). 

### 2.11. Microbiological Analysis—Total Aerobic Bacteria Count

The number of mesophilic bacteria in herb extracts was determined as follows: 1 mL of aqueous extract was taken aseptically and mixed with 9 mL of 0.9% NaCl. The sample was vortexed for 1 min and serial dilutions were made from 10^−1^ to 10^−9^ with 0.9% NaCl. The samples were thoroughly vortexed. Each dilution was directly inoculated on the PCA (Plate Count Agar) surface in duplicate. 0.1 mL of the respective suspensions were rubbed into the culture media. Bacterial colonies (CFU) numbers were read after 48 h incubation at 28 °C. Plates from two serial dilutions that grew from 15 to 150 colonies were selected for reading. The number of microorganisms was determined taking into account the dilution and the volume of inoculated material. The results were expressed as the log CFU per gram of each herb.2.12. Surface morphology- Scanning electron microscope (SEM) sample morphologies were analyzed using SEM/FIB Quanta 3D 200i (FEI) microscope. Before imaging, a 20 nm layer of Ag was sputtered on each sample, using Leica EM ACE600 High Vacuum Sputter Coater. Structure changes of plant material were verified at three different stages of the extraction process. The first scanning electron micrograph was made for ground herb that was not subjected to any procedure. The second one was made for herb plant material after extraction in water for 10 min at 70 °C and the third for samples subjected to cold plasma treatment for 8 min and subsequent extraction in water for 10 min at 70 °C. Samples after extraction were centrifuged for 5 min at 9000 rpm and filtered through Whatman paper. The remaining plant matter was collected and air-dried before further analysis.

### 2.12. Statistical Analysis

Data for statistical analysis was obtained for each method at least in three repetitions for each group. In the case of chemical analysis, six measurements were taken for each group. Statistical analysis of obtained data was performed using Statistica software version 13.3 (StatSoft, Tulsa, OK, USA). The normality of data distribution was tested using the Shapiro–Wilk test. Multifactorial ANOVA followed by the Tukey test was applied for analysis of the polyphenols, flavonoids and anthocyanins content. Similarities in antioxidant capacity of tested samples were verified by hierarchical clustering analysis performed using the Ward linkage method and the squared Euclidean as a distance measure. Microbiological contamination was analyzed with the usage of a *t*-test. The level of significance was set at α ≤ 0.05. In order to conduct a comparative analysis and to evaluate the chromatographic fingerprints of the volatile compounds, the AlfaSoft package with statistical quality control was used.

## 3. Results and Discussion

### 3.1. Color and pH

The effect of plasma treatment on color parameters of herb extracts is shown in Table 1. In general, there was no obvious effect of plasma on the color of herb extracts. In the case of one herb (*Ballota nigra*) all three-color parameters (L*, a*, b*) were affected by applied treatment. However, for most herbs, only one or two color parameters have changed. For instance, the change of L* color parameter was noted for *Sanguisorba officinalis*, a* for *Taraxacum officinale* and *Achillea millefolium*, b* for *Polygonum aviculare* and *Hypericum perforatum*. Two-color parameters have changed for *Andrographis paniculata* (L* and a*), *Leonurus cardiaca* (L* and b*), *Echinacea purpurea* and *Vaccinium myrtillus* (a* and b*). There were also two herb extracts (*Salvia officinalis* and *Urtica dioica*) the color of which have not changed after plasma treatment. It has been established that the color of plasma-treated samples is determined by the treatment conditions and biological status of herbs. Its change in herb extracts might be caused by pigment oxidation or degradation initiated by active species formed by plasma generators [6]. However, color may change also due to the higher extraction rate of bioactive compounds caused by the application of plasma treatment [15]. In turn, the pH of almost each herb extract was affected by the plasma similarly. All tested samples with the exception of *Sanguisorba officinalis* were characterized by the increased pH after applying plasma treatment (data not shown). The change of pH was quite similar for most of the extracts for instance *Echinacea purpurea* extract obtained with cold plasma had a pH higher of 0.25 in comparison to *control, Salvia officinalis* 0.22, *Polygonum aviculare* 0.11, *Vaccinium myrtillus* 0.16, *Taraxacum officinale* 0.1, *Hypericum perforatum* 0.14, *Achillea millefolium* 0.08, *Leonurus cardiaca* 0.11, *Ballota nigra* 0.1, *Andrographis paniculate* 0.27. However, an exceptionally high increase was observed *for Urtica dioica* of about 0.71. It has been reported that changes in pH of plasma-treated samples are dependent on its bioactivity and treatment method used [16]. The pH increase of the treated samples was quite unexpected taking into consideration that reactive nitrogen species (RNS) created by plasma can react with hydronium ions (H_3_O+) formed from water in a plasma generator. Both RNS and H_3_O+ may interact leading to the creation of nitric compounds, such as nitrous acid or nitric acid, which are supposed to decrease the pH of tested samples [17]. The opposite results may be caused by the intrinsic buffering capacity of the tested extracts [18].

### 3.2. Total Phenolic, Anthocyanins and Flavonoids Content

Polyphenols are the main class of secondary metabolites of plants. They are a large group of compounds widespread in nature, which include phenolic acids, flavonoids (flavonols, anthocyanins), tannins, lignans and stilbenes [19]. They are very important food components because of their antioxidant properties. In the experiment, we measured the content of total polyphenols as well as anthocyanins and flavonoids in control and plasma-treated herb extracts (Table 2). Plant extracts contained a wide range of polyphenols, from 9.96 ± 0.44 for control *Leonurus cardiaca* up to 79.46 ± 0.79 for plasma-treated *Vaccinium myrtillus*. In 11 out of 12 studied herb extracts a significant increase of total polyphenols content after the extraction process was observed, proceeded by the cold plasma application (8 min., 20 kHz). The polyphenol content in extracts increased by about 10% after plasma treatment. In the case of anthocyanins, the trend was inconclusive. Only four herb extracts exhibited higher content of anthocyanins after treatment. However, in those cases the rise was high. For instance, in *Polygonum aviculare,* there were about 77% more anthocyanins in relation to control, in *Salvia officinalis* 48%, in *Echinacea purpurea* 36% and in *Andrographis paniculata* 34%. In turn, in *Urtica dioica* extract, anthocyanins degraded completely after cold plasma application. In the remaining extracts (8 out of 12), there was not a significant difference in comparison to the control group. Similarly, flavonoids content increased only in four herb extracts. However, the increase was low (from 12% for *Urtica dioica* up to 21% for *Sanguisorba officinalis*). In one extract (*Hypericum perforatum*) flavonoid content dropped by about 4%, and the rest of the extracts were unaffected by the treatment. Previous studies reported that plasma treatment probably causes degradation of the cell membrane by immanent plasma reactive species, which enhance phenolic compound extraction into the intercellular space [20]. Moreover, in the liquid environment, the penetration of radicals is equal in the whole volume [10]. In our study, fragmented plant material was hydrated before plasma application. Thus, it could facilitate the extraction process. Plasma-based extraction can also contribute to the increase of phenolics by releasing them from glycosidic components or by degrading larger polyphenols into smaller compounds. Obtained results are in line with studies performed by Sarangapani et al., [21] who found more phenolic compounds in plasma-treated parboiled rice flour. More polyphenols were also noted in tomato-based beverages subjected to cold plasma processing [22]. On the contrary Garofulić et al., [23] reported degradation of polyphenol compounds but they were affected by longer exposure to plasma treatment. Other studies showed no effect of plasma on the anthocyanin content that was linked by the authors with the voltage used for the plasma generator [24]. Hence, treatment time, as well as the potency of the plasma activated water and voltage, has an important role in the final content of bioactive compounds in plasma-treated food. Short plasma treatment leads to the breakdown of chemical bonds, the dissociation of agglomerates or particles, and consequently significant increases in anthocyanin and phenolic content. Thus, it can be stated that the process parameters of cold plasma treatment are fundamental in extracting polyphenols from food.

### 3.3. Antioxidant Activity

**Figure 1:**
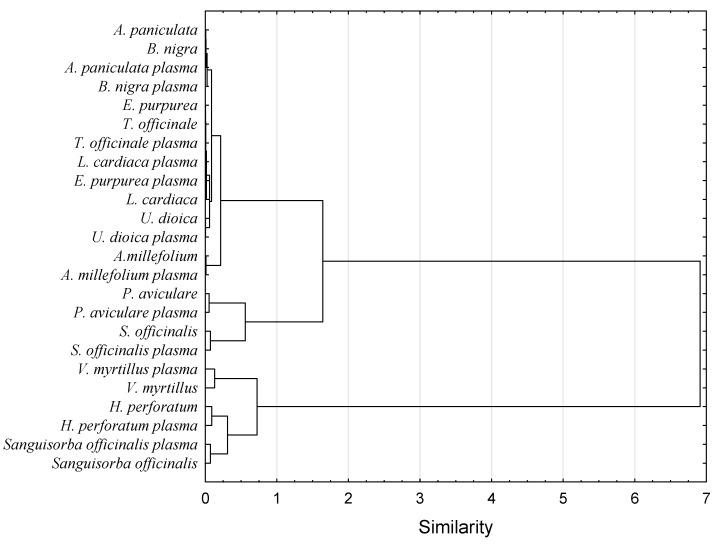
Comparison of antioxidant activity (measured by DPPH method) of plasma treated and non-treated water herb extracts.

**Figure 2:**
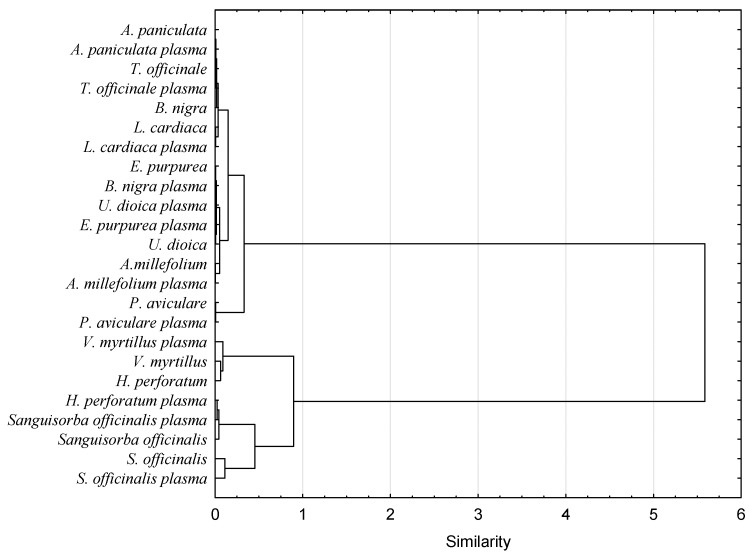
Comparison of antioxidant activity (measured by FRAP method) of plasma treated and non-treated water herb extracts.

**Figure 3:**
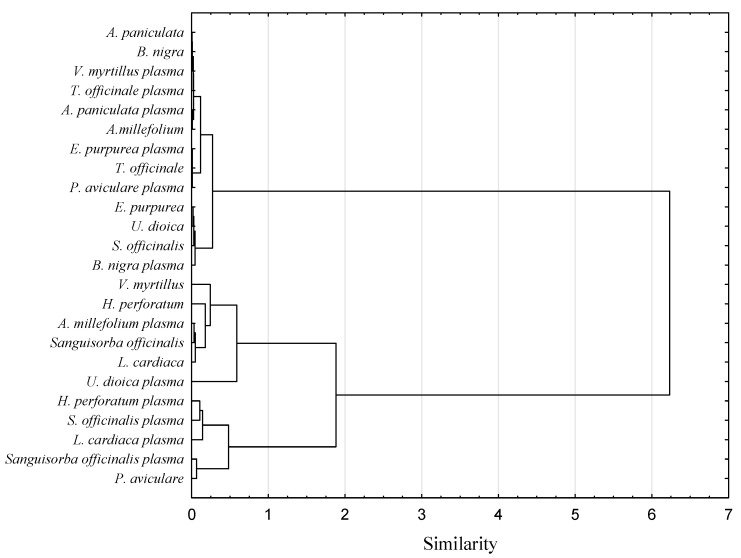
Comparison of antioxidant activity measured by the ABTS method of plasma-treated and non-treated water herb extracts.

The antioxidant capacity of food products is supposed to be measured using several different methods since those verify different action mechanisms of bioactive compounds and may exhibit synergistic reactions [25]. Thus, it gives more reliable information about the antioxidant activity of the tested material. The antioxidant capacity of herb extracts obtained in the study was assessed using metal reduction capacity (FRAP) and the scavenging capacity of organic radicals (DPPH and ABTS). Nine out of twelve herbs had significantly higher antioxidant capacity after plasma treatment measured by at least one method (DPPH, ABTS, or FRAP). In the case of one herb, it was noted that a little drop of antioxidant activity was measured by the ABTS method (*Sanguisorba officinalis*) after plasma application. Two extracts of *Polygonum aviculare* and *Andrographis paniculata* herbs were not observed to be affected by any of the treatments. *Vaccinium myrtillus* herb subjected to plasma treatment had the highest antioxidant capacity measured by DPPH, FRAP and ABTS methods (0.567, 0.404 and 0.596 M ascorbic acid/g dw, respectively). In turn, the lowest antioxidant activity was measured by the DPPH method for both *Urtica dioica* plasma-treated extract (0.005 M ascorbic acid/g dw) and by FRAP and ABTS methods for *Leonurus cardiaca* (0.021, 0.054, respectively) non-treated herb extracts. Obtained results are in line with literature data. Cold plasma treatment increased antioxidant activity in different food products, such as fresh-cut pears [26], cashew apple juice [27] or chili pepper [28]. However, there are also studies presenting no effect of cold plasma on the antioxidant activity of the food or even its reduction. Those differences were caused by the process parameters, mainly by the time of exposure to plasma [27]. In order to verify possible similarities in antioxidant capacity of tested samples, a hierarchical clustering analysis was performed using the Ward linkage method and the squared Euclidean as a distance measure. Dendrograms for each method analyzing antioxidant capacity (DPPH, FRAP and ABTS) were split into two clusters (Figure 1, Figure 2 and Figure 3). 

For both DPPH and FRAP analysis, herb distribution into two clusters was almost the same (except for *Salvia officinalis* herb). Meaning that these two methods investigated similar action mechanisms of bioactive ingredients. In turn, for the ABTS, the distribution of herbs was different. Moreover, in contrast to the previously described methods in the ABTS dendrogram, values of antioxidant capacities of herbs before and after plasma treatment were not placed close to each other. This observation points out that the ABTS method investigated a different spectrum of mechanisms responsible for the antioxidant capacity of the extracts in comparison to DPPH and FRAP methods. Previous studies proved that the correlation between DPPH and ABTS is strongly affected by the polarity of plant extracts [29]. Non-polar plant extracts exhibit a lower correlation between DPPH and ABTS activities. Furthermore, both these methods are complex methods covering multistep reaction mechanisms. Thus, it is hard to find a linear correlation between them.

### 3.4. Volatile Compounds Profile

**Figure 4:**
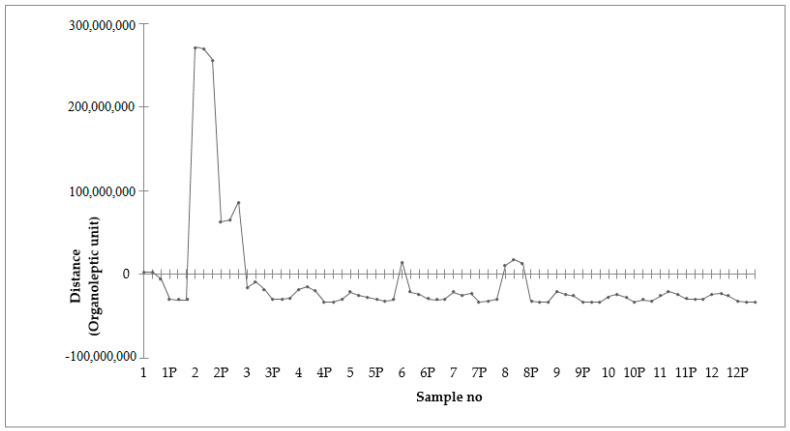
Chromatographic profiles of herb extracts non-subjected or subjected to cold plasma pretreatment. 1,1P: *Echinacea purpurea;* 2,2P: *Salvia officinalis*; 3,3P: *Urtica dioica*; 4,4P: *Polygonum aviculare*; 5,5P: *Vaccinium myrtillus*; 6,6P: *Taraxacum officinale*; 7,7P: *Hypericum perforatum*; 8,8P: *Achillea millefolium*; 9,9P: *Sanguisorba officinalis*; 10,10P: *Leonurus cardiaca*; 11,11P: *Ballota nigra*; 12,12P: *Andrographis paniculata*.

Based on the performed analysis we noted that 12 (for *Sanguisorba officinalis*) to 25 different volatile compounds were detected (for *Salvia officinalis* and for *Achillea millefolium*) depending on the species (Table 3). The most predominant volatiles recognized for almost every herb, both treated and non-treated with cold plasma was propanal, ethanol and methyl/ethyl formate. Propanal was noted in all tested samples with the exception of *Hypericum perforatum*. Methyl/ethyl formate was detected in 11 out of 12 herbs as well. The occurrence of this substance was not confirmed only in *Echinacea purpurea*. In turn, ethanol was noted in each cold plasma treated herb and almost in each non-treated herb (with the exception of *Echinacea purpurea).* In the case of six herbs, we observed a change of volatile compound profile after applying cold plasma treatment (*Echinacea purpurea,* *Salvia officinalis*, *Urtica dioica*, *Polygonum aviculare*, *Taraxacum officinale*, *Hypericum perforatum*). Plasma treated herb extracts contained fewer kinds of volatile compounds compared to untreated samples. Figure 4 shows a statistical quality control graph of differences in volatile compound profiles between herb extracts obtained with the application of cold plasma treatment and extracts acquired without treatment. Presented data reflect odor differences between samples measured using Euclidian distance. It can be clearly observed that the samples exposed to the plasma had fewer volatile compounds detected in comparison to untreated samples, which resulted in a less complex profile of volatile compounds in those samples in comparison to control groups. Thus, it can be concluded that the intensity of the odor of the extracts after plasma treatment decreased. The same effect of plasma on the reduction of the volatile compound profile was observed for lemon verbena leaves treated with plasma for 5 min [30]. Plasma treatment may disintegrate the cellular structures in the same manner as in the case of microbes [6]. However, it increases the yield of volatiles in samples but also accelerates their evaporation [31]. Thus, the longer time of CP treatment led to a less pronounced odor of extracts. However, the outcome depends deeply on plant species, cold plasma process parameters, duration of the treatment and composition of the volatiles that occurred in the plant [32]. Reducing the intensity of the odor may be beneficial for the food industry in the case of herbs that have an intense smell but high biological activity.

### 3.5. Total Aerobic Bacteria Count

Obtained data showed that the extracts acquired from dry herbs were heavily contaminated by aerobic bacteria (Table 4). Most of the tested samples exceeded the upper permissible limit of bacterial count (>10^5^ cfu∙g^−1^). The performed study confirms literature reports that herbs available on the market are highly contaminated by microorganisms [33,34]. Cold plasma pre-treatment showed a germicidal property for most extracts. Nine out of twelve extracts subjected to the cold plasma procedure had fewer bacteria than the control group. Recently, a lot of research has been carried out on the effect of cold plasma on the reduction of microorganisms in food products. Most of them confirmed the antimicrobial properties of cold plasma [6,35]. It was proven that plasma-induced radicals attack the chemical structures of cell membranes which in consequence causes its death [36]. Depending on herb species, the number of aerobic bacteria decreased from 1.27 logCFU∙g^−1^ up to 3.48 logCFU∙g^−1^. The average bacteria content decrease, for each herb that plasma exerted antimicrobial property, was 2.58 logCFU∙g^−1^. For comparison, Hemmati et al. [6] noted a 1.5 logCFU∙g^−1^ drop of bacteria content in plasma-treated turmeric powder. Furthermore, Kim et al. [35] observed that the number of aerobic bacteria in black pepper powder and red pepper powder was reduced by plasma application of about 2.3 logCFU∙g^−1^ and 0.6 logCFU∙g^−1^, respectively. We noted a higher ability of plasma treatment on bacteria elimination, probably because the herbs were suspended in water before plasma treatment. Water ensures the stability of reactive oxygen species. Additionally, plasma-activated water has higher fluidity than plasma itself, which makes the decontamination process more effective [37]. However, there was no observed germicidal effect of plasma in the case of three herbs: *Urtica dioica, Vaccinium myrtillus* and *Ballota nigra.* This might occur from the different compositions of those herbs. It was previously reported that the presence of ions and ionic components in solutions protects enzymes and proteins from reactive species generated by plasma action [38,39].

### 3.6. Surface Morphology Analysis

**Figure 5:**
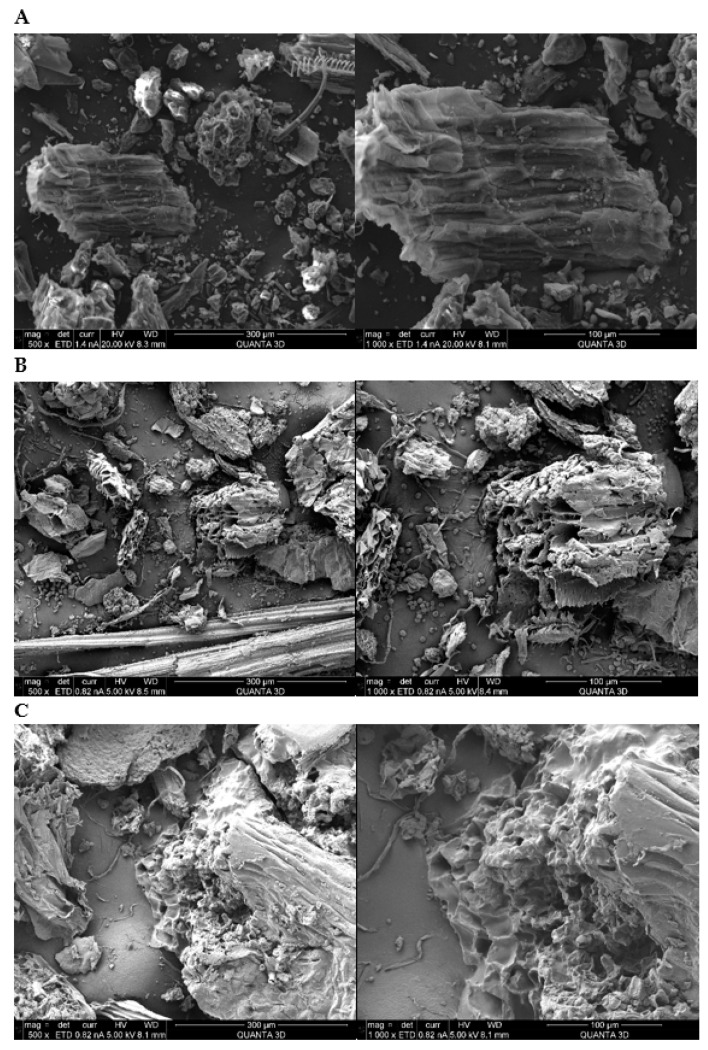
SEM micrographs of *Sanguisorba officinalis* magnified 500× (left column) and 1000× (right column): untreated ground herb (**A**), extracted without previous treatment (**B**), extracted with cold plasma treatment (**C**).

SEM technology was used to analyze the impact of cold plasma pretreatment on the surface morphology of ground herbs. Surfaces of both water extracted herb (B) and herb pretreated with cold plasma and extracted with water (C) looks visibly violated (Figure 5). The structures were damaged greatly in comparison to control ground herb (A). At the very first sight, it seems that the herb extracted just in water for 10 min exhibited the greatest visible external injuries. However, if we take a closer look, we can observe that the structure of the plant material subjected to previous cold plasma treatment is damaged more. There are no sharp edges on the surface, as in material from control group, because those edges were kind of melted and some sections were even completely detached. Our findings are in line with those of Zhang et al. [28] who observed the impact of cold plasma application on the surface of chili peppers. Grzegorzewski et al. [40] also noted the erosion of lamb’s lettuce induced by cold plasma. Additionally, Moritz et al. [41] who investigated the impact of cold plasma on the surface change of various materials, noted its effect on some of them, manifesting through the materials’ oxidation and shift of the free surface energies. Plasma caused the occurrence of roughness and a rusty crust of stainless steel. Moreover, the changed surface appearance accompanied with atomic concentration and higher polar fractions of free surface energy were found for rigid polyvinyl chloride, polypropylene and glass after plasma application. 

## 4. Conclusions

Cold plasma used on herbs suspended in water increased antioxidant activity in most of the extracts. The structure changes of the plant material caused by the treatment helped to extract more bioactive compounds in comparison to the control group. Furthermore, the composition of volatile compounds was reduced by plasma application meaning that the odor of those extracts was less intense. It is a good outcome taking into consideration, that most of the extracts at primary versions (before extraction) had strong scents. High-intensity odors may be a negative feature for extracts, which are intended to be used as food additives. Moreover, plasma exhibited germicidal properties, which is of key importance for non-thermal technology especially for extracting herbs, which are usually of poor microbiological quality. However, the impact of plasma was not equally significant for each herb, which was probably caused by the primary structure of the herbs and its chemical composition. To overcome those differences, the change of process parameters, selected individually for each herb might be the best solution. To summarize, cold plasma pre-treatment may be successfully introduced as a part of technological lines used for the extraction of plant materials, operating at low temperatures for a short period of time. Thus, cold plasma technology may be implemented into lines built up to meet the standards of green extraction chemistry.

## Figures and Tables

**Table 1:** The effect of cold plasma treatment on color parameters of water herbs extracts presented as mean values with standard deviations.

Herb Species	L*		a*		b*	
	Control	P-Treated	Control	P-Treated	Control	P-Treated
*Echinacea purpurea*	23.05 ± 0.56	24 ± 0.53	4.2 ± 0.3 a	7.88 ± 0.37 b	4.95 ± 0.32 a	7.88 ± 0.76 b
*Salvia officinalis*	31.79 ± 0.37	32.23 ± 0.55	17.06 ± 0.39	17.05 ± 0.33	20.62 ± 0.48	21.2 ± 0.76
*Urtica dioica*	21.03 ± 0.4	20.89 ± 0.49	-0.05 ± 0.1	0.07 ± 0.12	2.05 ± 0.11	2.35 ± 0.13
*Polygonum aviculare*	61.4 ± 1.33	60.42 ± 0.9	1.09 ± 0.08	1.42 ± 0.1	21.98 ± 0.76 a	24.16 ± 0.44 b
*Vaccinium myrtillus*	61.23 ± 2.16	58.49 ± 1.47	0 ± 0.07 a	2.08 ± 0.42 b	27.74 ± 2.39 a	34.05 ± 0.24 b
*Taraxacum officinale*	26.13 ± 0.65	25.64 ± 0.67	14.28 ± 0.56 a	15.15 ± 0.86 b	9.76 ± 0.34	10.41 ± 0.67
*Hypericum perforatum*	47.28 ± 0.3	47.74 ± 0.56	8.93 ± 0.18	8.8 ± 0.22	21.55 ± 0.48 a	25.87 ± 0.19 b
*Achillea millefolium*	33.35 ± 0.55	32.63 ± 0.48	15.19 ± 0.29 a	16.55 ± 0.32 b	22.44 ± 0.5	21.93 ± 0.75
*Sanguisorba officinalis*	49.77 ± 0.72 a	51.17 ± 0.45 b	8.94 ± 0.38	8.52 ± 0.24	35.33 ± 0.65	36.67 ± 0.47
*Leonurus cardiaca*	36.28 ± 0.43 a	37.57 ± 0.43 b	16.82 ± 0.63	16.52 ± 0.48	26.2 ± 1 a	28.17 ± 1.42 b
*Ballota nigra*	31.16 ± 0.45 a	32.41 ± 0.38 b	17.41 ± 0.88 a	18.57 ± 0.16 b	17.95 ± 1.15 a	21.74 ± 0.49 b
*Andrographis paniculata*	35.21 ± 0.43 a	33.13 ± 0.74 b	-0.49 ± 0.11 a	2.02 ± 0.68 b	18.2 ± 2.39	19.6 ± 1.01

Mean values bearing different letters (a, b) within each raw for each parameter differ significantly (*p* < 0.05).

**Table 2:** The effect of cold plasma treatment on the content of total polyphenols, anthocyanins and flavonoids in water herb extracts. Data presented as mean values with standard deviations.

Herb Species	Total Polyphenols [mg Gallic Acid/g DW]		Anthocyanins [mg Gallic Acid/g DW]		Flavonoids [mg Gallic Acid/g DW]	
	Control	P-Treated	Control	P-Treated	Control	P-Treated
*Echinacea purpurea*	17.26 ± 1.57 a	19.34 ± 0.76 b	9.8 ± 1.43 a	13.36 ± 1.98 b	3.04 ± 0.08	3.15 ± 0.24
*Salvia officinalis*	40.35 ± 1.22 a	43.64 ± 1.05 b	20.39 ± 1.51 a	30.31 ± 2.52 b	8.81 ± 1.09 a	10.28 ± 0.25 b
*Urtica dioica*	18.01 ± 0.78	17.33 ± 0.51	89.47 ± 10.26 a	0 ± 0 b	4.04 ± 0.25 a	4.54 ± 0.09 b
*Polygonum aviculare*	19.96 ± 1.41 a	21.88 ± 0.74 b	4.42 ± 0.3 a	7.85 ± 0.88 b	2.86 ± 0.07	2.8 ± 0.03
*Vaccinium myrtillus*	74.39 ± 1.05 a	79.46 ± 0.79 b	0 ± 0	0 ± 0	5.47 ± 0.84 a	6.49 ± 0.07 b
*Taraxacum officinale*	14.43 ± 0.35 a	16.02 ± 0.8 b	11.48 ± 0.93	11.55 ± 3.21	3.24 ± 0.25	3.45 ± 0.05
*Hypericum perforatum*	56.95 ± 1.81 a	61 ± 0.69 b	21.33 ± 2.99	21.96 ± 1.55	12.05 ± 0.13 a	11.53 ± 0.16 b
*Achillea millefolium*	19.11 ± 0.57 a	21.26 ± 0.35 b	13.81 ± 1.38	12.44 ± 3.14	4.66 ± 0.08	4.72 ± 0.11
*Sanguisorba officinalis*	42.87 ± 2.15 a	47.75 ± 0.77 b	17.5 ± 1.47	15.95 ± 3.04	4.77 ± 0.11 a	5.8 ± 0.12 b
*Leonurus cardiaca*	9.96 ± 0.44 a	11 ± 0.7 b	0.22 ± 0.27	1.11 ± 1.22	1.86 ± 0.06	1.84 ± 0.02
*Ballota nigra*	12.99 ± 0.64 a	15.05 ± 0.33 b	0 ± 0	0 ± 0	2.35 ± 0.04	2.28 ± 0.03
*Andrographis paniculata*	10.63 ± 0.42 a	11.7 ± 0.37 b	10.51 ± 1.23 a	14.15 ± 0.81 b	2.18 ± 0.05	2.14 ± 0.03

Mean values bearing different letters (a, b) within raw for each parameter differ significantly (*p* < 0.05).

**Table 3:** Volatile compounds identified in herb extracts non-treated or treated with cold plasma.

Compound	DB5	DB1701	1	1P	2	2P	3	3P	4	P	5	5P	6	6P	7	7P	8	8P	9	9P	10	10P	11	11P	12	12P
Methyl/ethyl formate	389	471			+	+	+	+	+	+	+	+	+	+	+	+	+	+	+	+	+	+	+	+	+	+
Acetaldehyde	418	497			+		+		+	+	+	+	+	+	+	+	+	+	+	+	+	+	+	+	+	+
Ethanol	443	536 554		+	+	+	+	+	+	+	+	+	+	+	+	+	+	+	+	+	+	+	+	+	+	+
Diethyl eter	477	535															+	+								
Pentane	500	500													+											
Propanal	504	607	+	+	+	+	+	+	+	+	+	+	+	+			+	+	+	+	+	+	+	+	+	+
2-methylpropanal	515	617													+	+										
2-mercaptoethanol	546	890																								
1-propanol	548	649												+												
Butanal	565	663													+	+										
Formic acid	586	777			+																				+	+
1-propanol, 2-methyl-	615	749															+	+								
Ethyl acetate	616	695			+	+	+	+	+	+			+	+							+	+	+	+	+	+
Acetic acid	623	760																								
2-methylbutanal	685	728															+	+			+	+	+	+		
Isopropyl acetate	650	729																								
Pentanal	686	779					+	+			+	+														
Acetoin	687	868			+	+																				
Pentan-2-one	689	763								+									+	+						
Ethyl propanoate	716	762											+	+	+	+	+	+			+	+	+	+	+	+
Propyl acetate	717	804									+	+														
2- ethyl furan	718	729			+	+		+	+	+																
Butanethiol	745	750				+	+	+	+	+	+	+	+				+	+	+	+	+	+				
Ethyl isobutyrate	746	824	+	+	+	+	+		+		+	+			+	+	+	+					+	+	+	+
Hexenal	784	890	+	+			+	+			+	+	+			+	+	+			+	+				
Cyclopentanone	794	907 917						+						+												
Butanoic acid	803	970															+	+			+	+	+	+		
Ethyl 2-methylbutyrate 3-hexanol	823	929									+	+														
Furfural	824	949															+	+								
Ethyl isovalerate	854	927			+	+	+	+																		
3-methylbutanoic acid	855	1023															+	+								
Pentanoic acid	889	1065			+	+									+	+										
2-heptanone	891	1011					+	+																		
3-heptanone	897	961															+	+								
Nonan	900	900			+	+					+	+	+		+	+	+	+	+	+					+	+
Heptanal	901	979							+	+											+	+	+	+		
Alpha-pinene	935	963	+	+	+	+	+		+	+	+	+	+		+	+	+	+								
Benzaldehyde	965	1057	+										+	+												
Beta-pinene	956	979					+		+	+															+	+
Sabinene = Thujene	959	1000	+		+	+					+	+					+	+								
1-octen-3-one	979	1061											+				+	+								
Butyl butanoate	981	1062																	+	+	+	+			+	+
Myrcene	991	1025			+	+																			+	+
Z-3-hexen-1-ol acetate	1001	1084			+	+	+	+			+	+					+	+			+	+	+	+		
Beta-pinene	1004	991	+	+			+		+	+			+	+	+	+										
Alpha-terpinene	1022	1081							+	+	+	+			+		+	+			+	+	+	+		
Formic acid	1023	1122					+	+																		
E-3-octen-2-one	1026	1124			+	+							+	+												
Alpha-phellandrene	1027	1009	+	+							+	+							+	+					+	+
2-octenal	1036	1135				+							+				+	+			+	+	+	+		
L-limonene Limonene	1056	1060	+	+					+																+	+
1-octenal	1071	1176			+		+	+											+	+			+	+		
Propyl hexanoate	1073	1172	+	+							+	+									+	+				
P-cymenene	1085	1170				+	+	+	+				+								+	+	+	+	+	+
Nonan-2-one 3-nonenal	1088	1200									+	+					+	+	+	+						
Linalool	1091	1095			+	+	+																			
Terpinolene	1102	1123			+		+	+						+	+	+										
Fenchol	1118	1226									+	+			+											
Limonene oxide	1132	1193														+										
Maltol	1134	1286																			+	+	+	+		
Citronellal	1163	1240				+									+											
Camphor	1181	1293																	+	+						
Trans carveol	1193	1367			+																					
Terpinen-4-ol Alpha-terpineol	1196	1271				+	+																			
(E)-carveol	1197	1372												+		+										
Propyl heptanoate	1226	1252			+																					
Geranial	1258	1438																			+	+				
P-menthadienhydro-peroxide	1343	1514																			+	+				
Gamma-nonalactone	1347	1577	+	+													+	+								
Eugenol	1361	1526			+		+	+													+	+				
Trans-2-undecenal	1375	1503													+											
Methyl eugenol	1412	1618			+	+																				
Vanilin	1426	1697													+	+										
Methyl cinnamate	1428	1524	+																							
Isoeugenol	1455	1658			+																					
Delta-decalactone	1467	1745													+											
Isoeugenol	1479	1637	+	+											+	+										
Beta-caryophyllene	1501	1514																			+	+				
Ethyl cinnamate	1504	1612	+	+																						
Methyl dodecanoate	1524	1624				+																				
Cumarin	1527	1709	+	+													+	+								
Isopropyl cinnamate	1555	1654		+																						
Cis-3-hexenyl benzoate	1559	1697				+																				

1,1P: Echinacea purpurea; 2,2P: Salvia officinalis; 3,3P: Urtica dioica; 4,4P: Polygonum aviculare; 5,5P: Vaccinium myrtillus; 6,6P: Taraxacum officinale; 7,7P: Hypericum perforatum; 8,8P: Achillea millefolium; 9,9P: Sanguisorba officinalis; 10,10P: Leonurus cardiaca; 11,11P: Ballota nigra; 12,12P: Andrographis paniculata.

**Table 4:** Total number of aerobic bacteria in untreated herb extracts and in extracts obtained with previous cold plasma treatment.

Herb Species	Total Aerobic Bacteria Count [log10 CFU/g]	
	Non-Treated	Plasma Treated
*Echinacea purpurea*	8.53 ± 0.06 a	6.13 ± 1.75 b
*Salvia officinalis*	8.09 ± 0.02 a	4.93 ± 0.02 b
*Urtica dioica*	9.1 ± 0.61	9.19 ± 0.79
*Polygonum aviculare*	8.46 ± 0.06 a	5.73 ± 0.01 b
*Vaccinium myrtillus*	2.23 ± 1.97	1.0 ± 1.73
*Taraxacum officinale*	7.07 ± 0.72 a	4.61 ± 0.13 b
*Hypericum perforatum*	4,78 ± 0.42 a	3.05 ± 0.02 b
*Achillea millefolium*	8.81 ± 0.2 a	5.59 ± 4.84 b
*Sanguisorba officinalis*	8.63 ± 0.07 a	5.8 ± 0.14 b
*Leonurus cardiaca*	10.05 ± 0.1 a	6.57 ± 0.11 b
*Ballota nigra*	7.7 ± 0.26	1.33 ± 2.31
*Andrographis paniculata*	4.91 ± 0.18 a	3.64 ± 0.08 b

Mean values bearing different letters (a, b) within raw differ significantly (*p* < 0.05).

## Data Availability

All the data is available within the article.
